# Biodegradable hollow mesoporous organosilica nanotheranostics (HMONs) as a versatile platform for multimodal imaging and phototherapeutic-triggered endolysosomal disruption in ovarian cancer

**DOI:** 10.1080/10717544.2021.2021322

**Published:** 2021-12-30

**Authors:** Pengfei Li, Bingquan Lin, Zhian Chen, Pan Liu, Jiaqi Liu, Weili Li, Ping Liu, Zhaoze Guo, Chunlin Chen

**Affiliations:** a Department of Obstetrics and Gynecology, Nanfang Hospital, Southern Medical University, Guangzhou, China; b Department of Medical Imaging Center, Nanfang Hospital, Southern Medical University, Guangzhou, China; c First Clinical Medical College, Southern Medical University, Guangzhou, China; d Breast Center, Department of General Surgery, Nanfang Hospital, Southern Medical University, Guangzhou, China

**Keywords:** Hollow mesoporous organosilica nanoparticles (HMONs), ovarian cancer, multimodal imaging, phototherapy, lysosomal disruption

## Abstract

A major impediment in the development of nanoplatform-based ovarian cancer therapy is endo/lysosome entrapment. To solve this dilemma, a hollow mesoporous organosilica-based nanoplatform (HMON@CuS/Gd_2_O_3_) with a mild-temperature photothermal therapeutic effect and multimodal imaging abilities was successfully synthesized. HMON@CuS/Gd_2_O_3_ exhibited an appropriate size distribution, L-glutathione (GSH)-responsive degradable properties, and high singlet oxygen generation characteristics. In this study, the nanoplatform specifically entered SKOV-3 cells and was entrapped in endo/lysosomes. With a mild near infrared (NIR) power density (.5 W/cm^2^), the HMON@CuS/Gd_2_O_3_ nanoplatform caused lysosome vacuolation, disrupted the lysosomal membrane integrity, and exerted antitumour effects in ovarian cancer. Additionally, our *in vivo* experiments indicated that HMON@CuS/Gd_2_O_3_ has enhanced T1 MR imaging, fluorescence (FL) imaging (wrapping fluorescent agent), and infrared thermal (IRT) imaging capacities. Using FL/MRI/IRT imaging, HMON@CuS/Gd_2_O_3_ selectively caused mild phototherapy in the cancer region, efficiently inhibiting the growth of ovarian cancer without systemic toxicity *in vivo*. Taken together, the results showed that these well-synthesized nanoplatforms are likely promising anticancer agents to treat ovarian cancer and show great potential for biomedical applications.

## Introduction

The incidence rate of ovarian cancer ranks third among female reproductive system malignant tumors, but the mortality rate ranks first (Bray et al., 2018; Sung et al., 2021). Because the clinical symptoms of early-stage ovarian cancer are atypical and lack specificity, early-stage tumors are challenging to detect, and 70% of ovarian cancers are diagnosed as stage III or IV. Ovarian cancer is also associated with a poor prognosis; approximately 70% of patients have a survival time of less than five years, and approximately 70% of patients have tumors that relapse within three years after surgery (Jayson et al., 2014; Lheureux et al., 2019). Therefore, novel strategies for diagnosis and treatment are needed to improve the survival of patients with ovarian cancer.

The lysosome is a crucial organelle – the digestive organ in cells (Zhu et al., 2017; Nakamura et al., 2019). Because of their strong degradation ability, lysosomes can remove cytotoxic substances, damaged or dead organelles, and mismatched proteins to maintain intracellular homeostasis (Mukherjee et al., 2019). Lysosomes also regulate intracellular signal transduction pathways. When the tumor microenvironment is hypoxic and undernourished, an alternative energy source is rapidly provided by degrading and recycling cellular components through lysosomes to meet the needs of cell growth and proliferation (Zhu et al., 2021). Therefore, tumor development and progression are closely related to the function of lysosomes, and triggering lysosome damage may be an effective method to induce tumor cell death.

Nanomedicines have recently been widely used in cancer therapy (Chen et al., 2021; Guo et al., 2021). Most nanoparticles (NPs) will be phagocytized into lysosomes after entering tumor cells through the endosomal pathway (Guo et al., 2018; Jin et al., 2021). Therefore, many scholars have developed NPs with ‘lysosomal escape’ to improve the concentration of drugs in the cytoplasm (Zhang et al., 2018, 2020). Additionally, several studies have validated that photosensitive NPs are an effective way to destroy the lysosome membrane induced by near infrared (NIR) irradiation, promoting NPs to escape from lysosomes and then playing an effective therapeutic role in the cytoplasm (Jiang et al., 2020; Shi et al., 2020). Therefore, we developed smart NPs that could directly damage lysosomes under NIR irradiation to achieve good antitumour effects.

NIR-based phototherapy (PT) has attracted more attention for the targeted treatment of malignant tumors because of the advantages of minimal harm to normal tissues, noninvasiveness, and efficient therapeutic ability. The combination of mild photothermal therapy (PTT) and photodynamic therapy (PDT) can achieve much better therapeutic efficiency because mild hyperthermia can elevate the level of oxygen in the tumor because of the temperature increase in blood flow, thus overcoming hypoxia­associated resistance to PDT (Yu et al., 2021). Several Cu-based nanoparticles have shown good photothermal conversion efficiency and unique photodynamic capability under NIR laser light illumination (Li et al., 2018; Tang et al., 2020). Among them, mesoporous silica nanoparticles (MSNs) and porous hollow silica nanoparticles have been studied as drug delivery systems because of their good *in vitro* and *in vivo* biocompatibility (Jiang et al., 2021). Recently, the incorporation of disulfide bonds (–S–S–) into the silica framework has helped to achieve fast degradation of organic/inorganic hybrid nanoparticles through intracellular glutathione (GSH) stimulation (Li et al., 2020; Wu et al., 2021). For example, Chen et al. (2020) reported a structure-dependent, GSH-responsive biodegradable, dendritic mesoporous organosilica nanoparticle that performed as an efficient delivery platform for therapeutic biomacromolecules in cancer treatment. Shi et al. identified an organic–inorganic hybridized hollow mesoporous organosilica nanoparticle (HMON) based on a ‘chemical homology’ mechanism for guest drug molecule encapsulations (Huang et al., 2017). Inspired by these studies, HMONs with a disulfide-bonded hybrid framework were selected as a model drug delivery system in our study. Different from other methods, CuS nanocrystals were grown *in situ* onto the surface of HMONs without the help of thiol groups, and HMON@CuS NPs were synthesized (Guo et al., 2020); these HMON@CuS NPs exhibited good photothermal conversion efficiency and better biocompatibility. However, the role of HMON@CuS NP-produced PTT&PDT in inducing lysosome damage in ovarian cancer remains unclear.

Furthermore, identifying and eliminating visible tumors is the main method to improve the prognosis and reduce the recurrence rate of ovarian cancer patients. However, completely removing the lesions in traditional surgery is challenging, particularly in patients with extensive peritoneal metastasis. NIR-based PTT and PDT are potential methods to eliminate microinvasive lesions. Using fluorescence and MR multimodality imaging, we can achieve real-time visualization of tumor identification and PT and identify an effective time window for PT intervention.

Herein, we attempted a novel theranostic nanoplatform by integrating DIR and Gd_2_O_3_ into HMON@CuS NPs to form the versatile platform HMON@CuS/Gd_2_O_3_ for MRI/NIR fluorescence multimodality imaging-guided PT. HMONs served as nanocarriers, CuS served as a photothermal agent, Gd_2_O_3_ served as an MRI contrast enhancer, and DIR served as an NIR fluorescence imaging agent. Therefore, HMON@CuS/Gd_2_O_3_ NPs can generate local hyperthermia and oxidative stress elevation at tumor lesions and achieve real-time visualization of tumor identification and elimination. After treatment with HMON@CuS/Gd_2_O_3_ under NIR irradiation, the *in vitro* and *in vivo* ovarian cancer proliferation abilities were evaluated. Additionally, lysosomal disruption was detected to explain the antitumour mechanism induced by HMON@CuS/Gd_2_O_3_. Thus, the multifunctional nanoplatform HMON@CuS/Gd_2_O_3_ might present great potential for precise cancer theranostics in ovarian cancer.

## Materials and methods

### Materials

Cetyltrimethylammonium chloride solution (CTAC), triethanolamine (TEA), tetraethoxysilane (TEOS), sodium citrate (3-mercaptopropyl)-trimethoxysilane (MPTES), concentrated HCl (37%), NH_3_•H_2_O, Na_2_S•9H_2_O, CuCl_2_•2H_2_O, Gd_2_Cl_3_•6H_2_O, 25 wt% bis [3-(triethoxysilyl)propyl]tetrasulfide (BTES), and NaOH were purchased from Sigma–Aldrich (MO, USA). The C18PMH-mPEG was purchased from Laysan Bio Inc. (AL, USA). PBS, DMEM, fetal bovine serum (FBS), and .05% trypsin-EDTA were obtained from Gibco (NY, USA). Human ovarian cancer cells (SKOV-3) were purchased from the cell bank of the Chinese Academy of Sciences (Shanghai, China).

#### Synthesis of HMON

According to a previous study, we performed an ammonia-assisted selective etching strategy to construct the hollow structure of HMON nanocarriers. Briefly, 20 mL of deionized water (ddH_2_O) was mixed with 2.1 mL of CTAC and 50 µl of TEA solution and stirred at 95 °C, and then 1 mL of TEOS was added dropwise to the mixed solution. One hour later, a mixture of BTES (1 mL) and TEOS (1 mL) was added dropwise and reacted for another 4 h. After that, the mixture was collected and washed with absolute ethanol, followed by stirring with HCl solution at 80 °C for 12 h. The mixture was further etched with ammonia solution at 60 °C for 3 h; finally, the HMON products were obtained after centrifugation.

#### Synthesis of HMON@CuS@Gd_2_O_3_

First, 30 mg of HMON products was stirred with 15 mg of CuCl_2_·6H_2_O in aqueous solutions. Six hours later, 30 mg of Na_2_S was added and stirred overnight at room temperature. After centrifugation and washing with ddH_2_O, the HMON@CuS NPs were collected and stored at 4 °C. To synthesize ultrasmall gadolinium oxide nanoparticles (Gd_2_O_3_), we first added 600 mg of gadolinium chloride hexahydrate into 10 mL of diethylene glycol (DEG). Subsequently, the mixture solution was vigorously stirred (750 rpm) at 80 °C. One hour later, 1.125 mL of aqueous NaOH solution (1 mmol/L) was added and stirred at 140 °C for 1 h, followed by stirring at 180 °C for 4 h; finally, ultrasmall Gd_2_O_3_ nanoparticles were obtained. After that, 5 mL of as-prepared Gd_2_O_3_ solution and 2 mL of as-prepared HMON@CuS solution (50 mg/mL) were mixed and sonicated at room temperature for 24 h. Finally, the products (HMON@CuS/Gd_2_O_3_) were obtained by centrifugation and washed with water several times.

#### Characterization of HMON@CuS/Gd_2_O_3_

The morphology of HMON@CuS/Gd_2_O_3_ was investigated using a transmission electron microscope (Jeol JEM-2000F, Tokyo, Japan). The particle size, size distribution, and zeta potential of the nanoparticles were measured using the dynamic light scattering assay (Zetasizer Nano ZS, Malvern, UK). The UV-visible absorption spectrum of HMON@CuS/Gd_2_O_3_ was measured using a spectrometer (Shimadzu UV-2600 UV, Kyoto, Japan). The morphologies of the PIH and PIGH NPs were examined using scanning electron microscopy (SEM; JEOL JSM-6301F, Tokyo, Japan). X-ray photoelectron spectroscopy (XPS) was performed using a PHI 5000 VersaProbe spectrometer and a monochromatic AlKα radiation source. Various concentrations of HMON@CuS/Gd_2_O_3_ in PBS solutions were irradiated using an 808 nm NIR laser (FS-Optics, Changchun, China), and the real-time temperatures were recorded using infrared thermography (FLIR E50 camera system, Shanghai, China).

#### The pH calculation of the photothermal conversion efficiency

Photothermal conversion efficiency of HMON@CuS/Gd_2_O_3_ was determined according to previous method. Detailed calculation was given as following:

(S1) $$\eta =\frac{hS(T_{\text{max}}-T_{\max,\mathrm{water}})-Q_{\text{dis}}}{I(1-10^{-A_{808}})}\times 100\%$$

(S2) $$Q_{\text{dis}}=\frac{C_{D}M_{D}(T_{\max(\text{water})}-T_{\mathrm{amb}})}{\tau_{S(\text{water})}}$$

(S3) $$\theta =\frac{t-T_{\mathrm{amb}}}{T_{\max}-T_{\mathrm{amb}}}$$

(S4) $$t=-\tau_{S}\times \;\text{ln}\;\theta$$

(S5) $$\tau_{S}=\frac{C_{D}M_{D}}{hS}$$

The photothermal conversion efficiency, *η*, is calculated using Equation (S1). The *T*_max_ means the equilibrium temperature, *T*_amb_ is ambient temperature of the surroundings. The Q_dis_ is heat loss from light absorbed by the container, and it is calculated by Equation (S2). *I* represents the incident laser power, ‘A808’ is the absorbance of samples at 808 nm. Where *h* means heat transfer coefficient, *S* represents the surface area of the container, and the *hS* is calculated from the Figure S(2B). The *hS* is calculated using the following Equation (S5).

### *In vitro* MR imaging

T1-weighted MR imaging of HMON@CuS/Gd_2_O_3_ (Gd^3+^ concentrations from .025 to .4 mM) was performed using a 3.0 T MRI scanner (Philips Ingenia 3.0 T, The Netherlands). The longitudinal proton relaxation times (T1) of HMON@CuS/Gd_2_O_3_ were measured using T1-weighted maps to calculate the relaxation rate.

#### Evaluation of the photothermal efficiency of HMON@CuS/Gd_2_O_3_

HMON@CuS/Gd_2_O_3_ solutions of 30, 60, 80, and 100 μg/mL were irradiated with an 808-nm NIR laser (FS-Optics, Changchun, China) at a power of .5 W/cm^2^ for 5 min. Blank PBS was used as a negative control. The temperature was recorded by infrared thermography (FLIR E50 Camera System, Shanghai, China). Additionally, the HMON@CuS/Gd_2_O_3_ solution of 80 μg/mL was irradiated with an 808 nm NIR laser (FS-Optics, Changchun, China) at a power of 1.0 W/cm^2^, .5 W/cm^2^, and .3 W/cm^2^ for 5 min, respectively.

#### Cell uptake of HMON@CuS/Gd_2_O_3_

SKOV-3 cells were seeded on 12-well plates (2 × 10^5^ cells/well) and cultured in DMEM containing 10% FBS and 1% penicillin-streptomycin solution (Gibco) in a humidified incubator (5% CO_2_ at 37 °C) for 24 h. The cells were then incubated with HMON@CuS/Gd_2_O_3_-containing medium (50 μg/mL) for 6 h and 24 h. Subsequently, the cells were observed by TEM and CLSM images to analyze the cellular uptake of HMON@CuS@Gd_2_O_3_.

#### In vitro phototherapeutic effect of HMON@CuS/Gd_2_O_3_

SKOV-3 cells were seeded on 96-well culture plates (6000 cells/well) and incubated in DMEM containing 10% FBS and 1% penicillin-streptomycin solution (Gibco) for 24 h. The cells were then incubated with fresh medium containing various concentrations of HMON@CuS/Gd_2_O_3_ for 6 h. Subsequently, the cells were washed twice with PBS and then irradiated at 808 nm at a power density of .5 W/cm^2^ for 5 min or without irradiation. After 24 h of incubation, cell viability was assessed using a standard Cell Counting Kit-8 (CCK-8) and LDH Assay Kit.

#### Intracellular distribution

SKOV-3 cells were seeded in 20 mm glass-bottomed dishes and incubated with HMON@CuS/Gd_2_O_3_-containing medium (50 μg/mL) for 0 h, 6 h, 12 h, and 24 h. Subsequently, the cells were washed with cold PBS and stained with LysoTracker Green DND-26 for 15 min and Hoechst 33342 for 10 min to label lysosomes and nuclei, respectively. Finally, the cells were washed with cold PBS and observed with CLSM. The morphology of lysosomes was observed in cells incubated with saline-containing medium or HMON@CuS/Gd_2_O_3_-containing medium with or without NIR irradiation for 5 min (808 nm, .5 W/cm^2^) using a similar procedure.

The morphology of lysosomes was also observed by TME. Briefly, SKOV-3 cells were seeded on six-well plates (4 × 10^5^ cells/well) and cultured in DMEM containing 10% FBS and 1% penicillin–streptomycin solution (Gibco) in a humidified incubator (5% CO_2_ at 37 °C) for 24 h. The cells were then incubated with HMON@CuS/Gd_2_O_3_-containing medium (50 μg/mL) for 6 h and treated with NIR irradiation for 5 min (808 nm; .5 W/cm^2^). The cells incubated with saline-containing medium and treated with NIR irradiation served as the blank control group. Finally, the cell samples were made into ultrathin sections, and the morphology of lysosomes was observed by TEM.

#### Measurement of intracellular ROS level

Briefly, cells were seeded in 24-well plates, and 24 h later, were HMON@CuS/Gd_2_O_3_ NPs added. The plates were subsequently treated with or without NIR irradiation. The cells were washed with PBS 24 h later and then sequentially stained with DCFH-DA reagent for 20 min, before imaging by fluorescence microscopy.

#### Integrity of the lysosomal membrane

Cells were plated in 24-well plates and cultured overnight. Twenty-four hours later, HMON@CuS/Gd_2_O_3_ NPs were introduced into SKOV-3 cells and cultured overnight. After PBS rinsing, the cells were treated with AO (5 µg mL^−1^) for 15 min and then rinsed with PBS. Next, the cells were observed under a fluorescence microscope, and the samples were excited at 488 nm. Emission was detected at 537 nm (green) and 615 nm (red).

#### *In vivo* multimodal imaging behaviors of HMON@CuS/Gd_2_O_3_

All animal experiments were conducted in accordance with the guidelines of the Institutional Animal Care and Use Committee (IACUC). The female nude mouse tumor model was constructed by the subcutaneous injection of SKOV-3 cells (2 × 10^6^ cells/mouse). When the tumor volumes reached 50–100 mm^3^, HMON@CuS/Gd_2_O_3_ NP solution was intravenously injected into the tumor-bearing mice. *In vivo* fluorescence imaging of tumor-bearing mice was conducted using a white-light and near-infrared dual-channel image-guided device (DIGITAL PRECISION MEDICINE Company, Beijing, China) at 6, 12, and 24 h after injection. For *ex vivo* fluorescence imaging, the tumor-bearing mice were sacrificed at 24 h, and the excised heart, liver, spleen, lung, kidney, brain, and tumor tissues were evaluated.

For *in vivo* MR imaging, SKOV-3 tumor-bearing mice were intravenously injected with HMON@CuS/Gd_2_O_3_ NP solution. T1-weighted MR images of the tumor area before and 24 h postinjection were collected using a 3.0 T Philips Ingenia MRI scanner with a special animal coil.

To perform *in vivo* IRT imaging, SKOV-3 tumor-bearing mice were also injected with HMON@CuS/Gd_2_O_3_ NP solution. Subsequently, the mice were irradiated with 808 nm irradiation at .5 W/cm^2^ for 8 min before and 24 h post-injection. The minimal tumor temperature changes during laser irradiation were monitored using an IR thermographic camera.

#### *In vivo* phototherapeutic effect of HMON@CuS/Gd_2_O_3_

SKOV-3 cells were collected and inoculated subcutaneously into the flanks of the right hind leg of mice. When the tumor size reached 50–100 mm^3^, various formulations were applied, such as saline with or without NIR, HMON@CuS/Gd_2_O_3_ with or without NIR irradiation (four mice/group), followed by 5 min of irradiation at the tumors (808 nm; .5 W/cm^2^) at 24 h post-injection. The length and width of the tumors were measured using a caliper. The corresponding tumor volume was calculated using the following formula: tumor volume (V) = length × width^2/^2. Finally, the mice were sacrificed, and the tumors were isolated to evaluate the therapeutic efficacy of different groups. The body weight of the mice was also monitored during treatments.

#### Statistical analysis

Metrological data were presented as means ± standard deviation (*n* ≥ 3). To compare two groups of data, Student's t-test (two-tailed) was used. To compare more than two groups of data, analysis of variance (ANOVA), followed by multiple comparisons of Tukey’s test was used. The statistical software used was SPSS 23.0 (SPSS, Inc., Chicago, IL, USA). *p* <.05 was considered statistically significant.

## Results and discussion

### Preparation and characterization of HMON@CuS/Gd_2_O_3_

**Figure 1.**
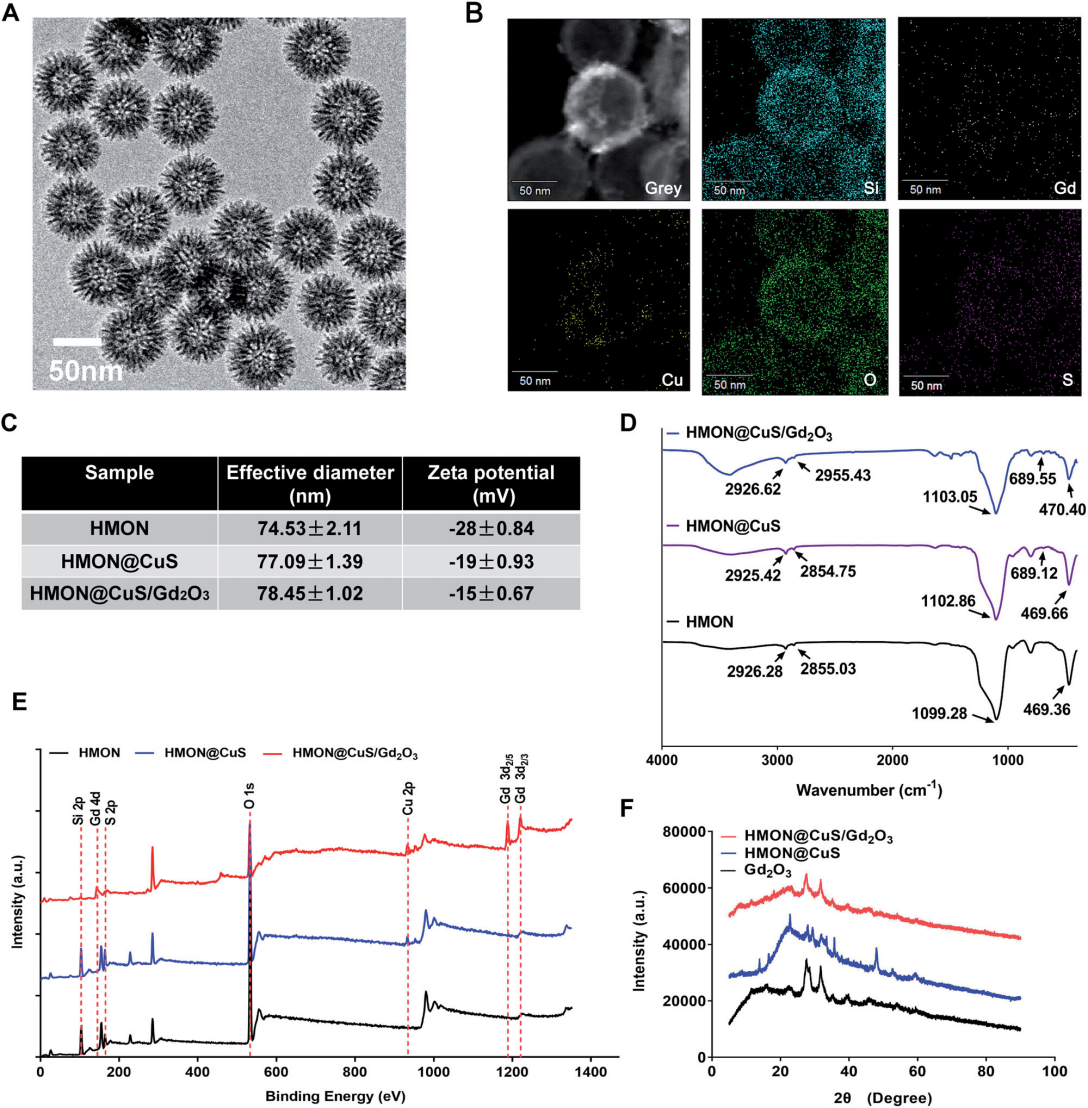
Characterization of HMON@CuS/Gd_2_O_3_. (A) Transmission electron microscopy (TEM) images of HMON@CuS/Gd_2_O_3_. Scale bar, 50 nm. (B) Element mapping images of HMON@CuS/Gd_2_O_3_. Scale bar, 50 nm. (C) Size distribution and zeta potential of HMON@CuS/Gd_2_O_3_ nanoparticles characterized by DLS. (D) Fourier transform infrared (FT-IR) spectra of HMON@CuS and HMON@CuS/Gd_2_O_3_ NPs. (E) X-ray photoelectron spectroscopy (XPS) of HMON and HMON@CuS/Gd_2_O_3_ NPs. (F) X-ray diffraction (XRD) patterns of HMON and HMON@CuS/Gd_2_O_3_ NPs.

HMON@CuS/Gd_2_O_3_ nanoparticles with uniform sizes of approximately 70 nm were synthesized (Figure 1(A)). Additionally, elemental mapping revealed that Si, Cu and Gd elements were distributed on the surface of these nanocomposites, but their contents in the central area were low, indicating that the nanocomposites had hollow structures (Figure 1(B)). DLS revealed that the diameter of HMONs was 74.53 ± 2.11 nm, while CuS and/or Gd_2_O_3_ loading led to a slight increase in the size of HMON@CuS (77.09 ± 1.39 nm) and HMON@CuS/Gd_2_O_3_ (78.45 ± 1.02 nm), and all of them exhibited a unimodal size distribution (Figure 1(C)). Additionally, the zeta potentials of HMON, HMON@CuS and HMON@CuS/Gd_2_O_3_ were approximately −28 ± .84 mV, −19 ± .93 mV and −15 ± .67 mV, respectively (Figure 1(C)). The mass ratio of Cu and Gd content in HMON@CuS/Gd_2_O_3_, as determined by inductively coupled plasma-atomic emission spectroscopy (ICP-AES), were almost 3:1. The FT-IR results revealed that HMON@CuS/Gd_2_O_3_ showed strong and broad peaks at 2926.62 cm^−1^ and 2955.43 cm^−1^ (attributed to -CH_2_ of C18PMH-mPEG chains) ( Liu et al., 2020), characteristic peaks at 1103.05 cm^−1^ and 470.40 cm^−1^ (attributed to -Si-O- of HMON), and a characteristic peak at 689.55 cm^−1^ (attributed to –Cu–S–) (He et al., 2018) (Figure 1(D)). The XPS results (Park et al., 2018) demonstrated that the HMON@CuS/Gd_2_O_3_ NPs possessed C (C 1 s peaks at 284.73 eV), O (O 1 s peaks at 532.23 eV), Si (Si 2p peaks at 102.58 eV), Cu (Cu 2p peaks at 932.68 eV), S (S 2p peaks at 169.88 eV) and Gd (Gd 4d peaks at 143.48 eV; Gd 3d peaks at 1187.98 eV and 1220.88 eV) (Figure 1(E)). Finally, the XRD results demonstrated the presence of a typical covellite crystalline phase of CuS and Gd_2_O_3_ on the HMON surface (Figure 1(F)). Taken together, these results consistently suggested the successful synthesis of HMON@CuS/Gd_2_O_3_.

#### Photothermal efficiency of HMON@CuS/Gd_2_O_3_

**Figure 2.**
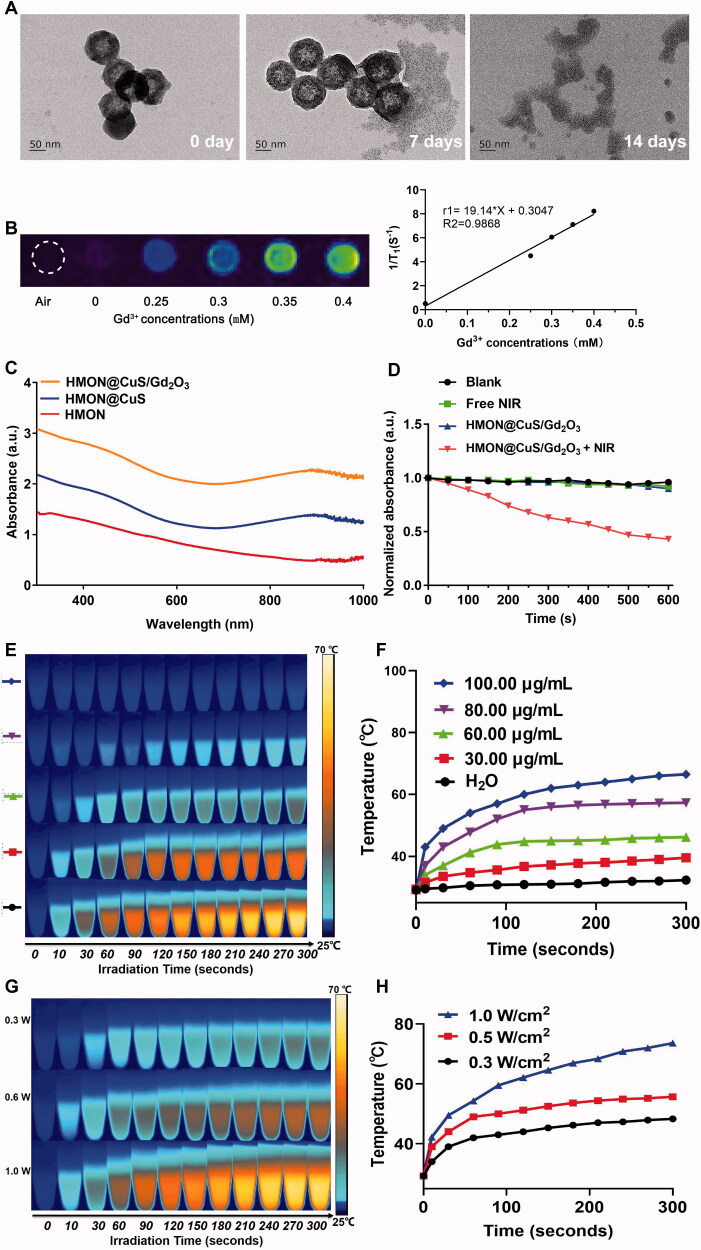
(A) TEM images of biodegradable HMON@CuS/Gd_2_O_3_ immersed in 10 mM GSH aqueous solution for 7 days and 14 days. (B) T1-weighted MRI images of HMON@CuS/Gd_2_O_3_ at various concentrations. (C) Ultraviolet Visible Spectroscopy (UV-Vis) spectrum. (D) Reactive oxygen species (ROS) production of HMON@CuS/Gd_2_O_3_ under NIR irradiation (.5 W/cm^2^; 5 min). (E&F) Temperature increase curve induced by different concentrations of HMON@CuS/Gd_2_O_3_ aqueous solution under NIR irradiation (1.0 W/cm^2^; 5 min). (G&H) Temperature increase curve induced by different NIR power intensities (.3 W/cm^2^, .5 W/cm^2^, and 1.0 W/cm^2^).

Because of the presence of disulfide bonds in the skeleton of HMONs, HMON@CuS/Gd_2_O_3_ NPs can be biodegraded in response to the high concentration of GSH (Hadipour Moghaddam et al., 2018; Li et al., 2020). TEM images revealed that when exposed to 10 mM GSH solution for 14 days, the completely hollow nanoparticles were hardly visible (Figure 2(A)). Similarly, the HMON@CuS/Gd_2_O_3_ were immersed into different concentration of GSH solution (0, 5, and 10 mM), respectively, and the degradation process was also monitored by ICP tests. As shown in the Figure S1, the Si ions release percentage reached approximately 28.2% dissolved in GSH solution (GSH = 10 mM), whereas only 15.9% was released in the absence of GSH after 60 h. Additionally, because of the presence of Gd_2_O_3_ (Zohdiaghdam et al., 2013; Fang et al., 2014), the as-prepared NPs were expected to be a promising MRI imaging contrast agent, a finding that was confirmed by *in vitro* MRI scanning (Figure 2(B)). UV–vis spectra results revealed that HMON@CuS and HMON@CuS/Gd_2_O_3_ both showed strong absorption in the NIR region, primarily attributed to the CuS nanocrystals (Figure 2(C)). Furthermore, the DPBF results revealed that HMON@CuS/Gd_2_O_3_ NPs plus NIR irradiation showed lower DPBF absorption, indicating that HMON@CuS/Gd_2_O_3_ had a strong photodynamic effect (Figure 2(D)). After NIR laser irradiation (.5 W/cm^2^) for 5 min, a dramatic temperature increase was observed in the HMON@CuS/Gd_2_O_3_ group, while no obvious temperature change was shown in the PBS group. The maximum increased temperature (Δ*T_max_*) of the HMON@CuS/Gd_2_O_3_ group (100 µg/mL) ∼37.5 °C, whereas the Δ*T_max_* of the other HMON@CuS/Gd_2_O_3_ groups (80 µg/mL, 60 µg/mL, and 30 µg/mL) increased to ∼29.9 °C, ∼17.0 °C, and ∼10.2 °C, respectively (Figure 2(E,F)). Furthermore, as the power density of NIR laser irradiation increased from .3 W/cm^2^ to 1.0 W/cm^2^, the maximum increased temperature (Δ*T_max_*) of HMON@CuS/Gd_2_O_3_ (80 µg/mL) increased from ∼19.0 °C to ∼44.2 °C (Figure 2(G,H)). Additionally, according to the linear regression curve between the cooling stage and negative natural logarithm of the driving force temperature of HMON@CuS/Gd_2_O_3_, its photothermal conversion efficiency was 34.2% (Figure S2). In total, these results demonstrated that HMON@CuS/Gd_2_O_3_ serves as a promising PTT, PDT, and functional MR imaging nanoparticle.

#### *In vitro* cellular uptake

**Figure 3.**
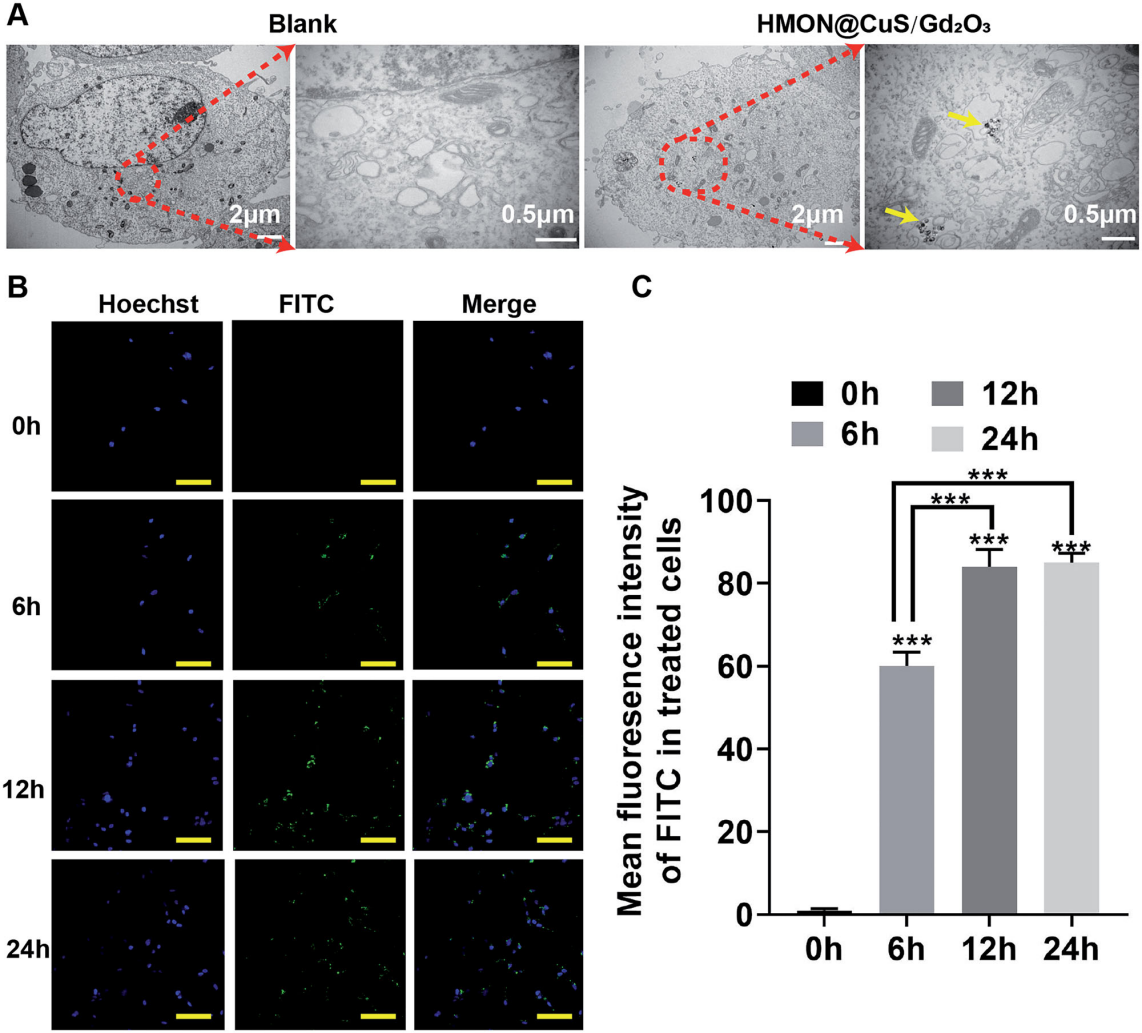
*In vitro* intracellular uptake detection. (A) TEM image of SKOV-3 cells; yellow arrows indicate nanoparticle locations. (B) CLSM images and fluorescence intensity profile analysis of SKOV-3 cells after treatment with FITC-labelled HMON@CuS/Gd_2_O_3_. Scale bar, 100 μm. *** *p* < .001.

FITC was encapsulated into HMON@CuS/Gd_2_O_3_, and the cellular uptake of HMON@CuS/Gd_2_O_3_ was then observed by transmission electron microscopy (TEM) and confocal laser scanning microscopy (CLSM). After incubation with HMON@CuS/Gd_2_O_3_ (50 μg/mL) for 6 h, TEM images showed hollow mesoporous nanotheranostics in the cytoplasm of SKOV-3 cells; however, this structure could not be found in the control group (Figure 3(A)). Consistently, the CLSM results indicated that the intracellular FITC fluorescence signal increases with increasing incubation time (Figure 3(B,C)). These obtained results revealed that HMON@CuS/Gd_2_O_3_ is internalized into SKOV-3 cells, supporting that NPs exert phototherapeutic and antitumour effects.

#### *In vitro* phototherapeutic effect of HMON@CuS/Gd_2_O_3_

**Figure 4.**
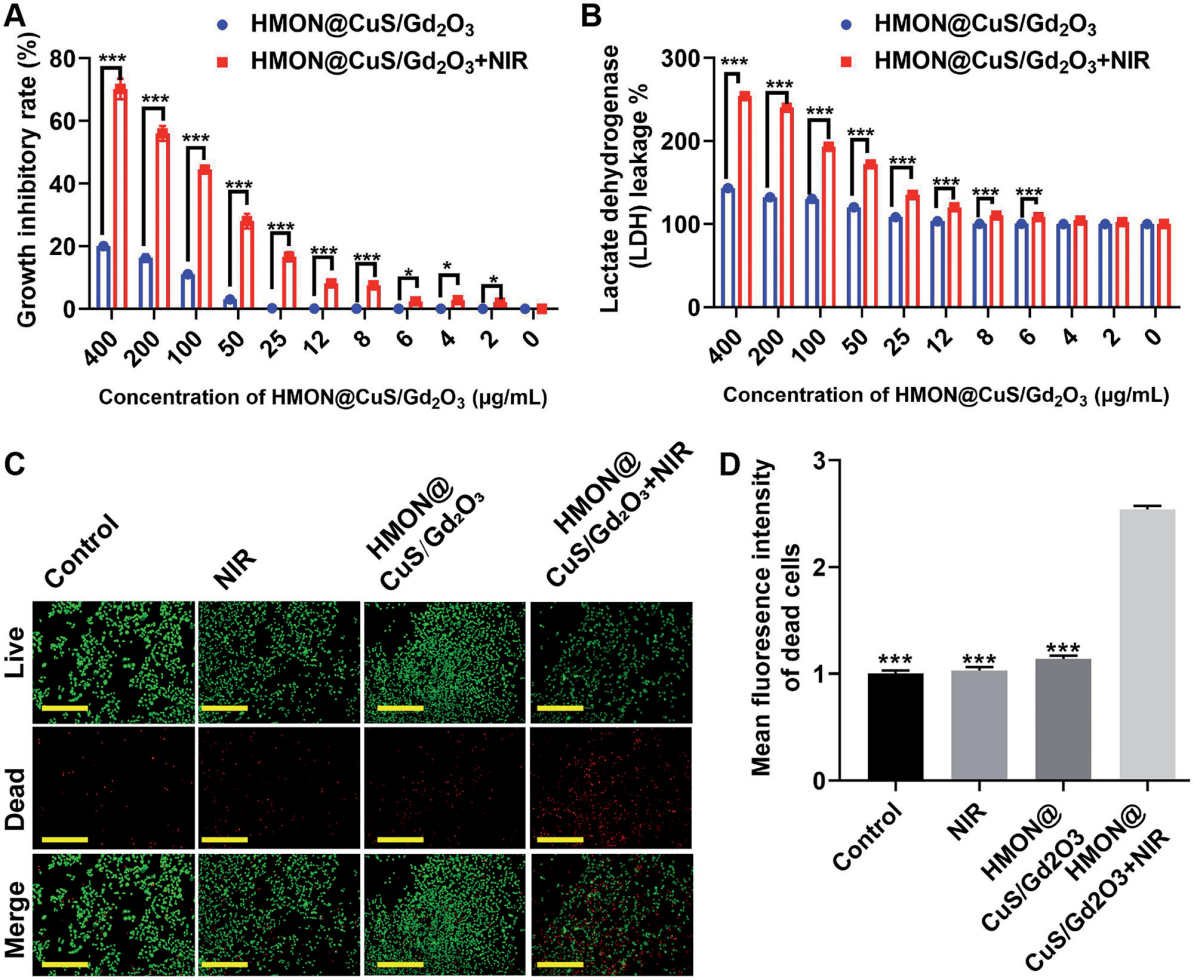
*In vitro* antitumour effect after treatment with HMON@CuS/Gd_2_O_3_ NPs. (A) Growth inhibitory rate of SKOV-3 cells detected by the CCK-8 assay. (B) LDH leakage of SKOV-3 cells after treatment with HMON@CuS/Gd_2_O_3_ plus NIR. (C&D) Live/dead assay and relative statistical analysis for SKOV-3 cells treated with HMON@CuS/Gd_2_O_3_ NPs with or without NIR irradiation (.5 W/cm^2^; 5 min) (Green: Live cells; Red: Dead cells). Scale bar, 150 μm. *p < .05, ***p < .001.

The *in vitro* phototherapeutic effects of HMON@CuS/Gd_2_O_3_ in the dark or under NIR irradiation were investigated in SKOV-3 cells based on the CCK-8 assay. First, SKOV-3 cells were incubated with HMON@CuS/Gd_2_O_3_ at different concentrations for 6 h and then were irradiated with an 808 nm laser at a power density of .5 W/cm^2^ for 5 min. Next, the antitumour therapeutic efficacies of NPs were tested using the CCK-8 assay after 24 h. The CCK-8 assay revealed that HMON@CuS/Gd_2_O_3_ in the dark did not induce significant changes in cell death. In the presence of NIR irradiation, the cell viability was dramatically decreased. At 12, 25, 50, 100, and 200 μg/mL of HMON@CuS/Gd_2_O_3_, the cell growth inhibitory rate in the dark was measured as ∼.13%, ∼.26%, ∼3.06%, ∼11.12%, and ∼16.20%, respectively, while the cell growth inhibitory rate under NIR irradiation increased rapidly to ∼8.17%, ∼16.7%, ∼28.05%, ∼44.53%, and ∼56.03%, respectively (Figure 4(A)). These results suggested that HMON@CuS/Gd_2_O_3_ provides a promising killing effect to ovarian cancer cells under NIR irradiation.

Consistently, the LDH assay revealed that LDH leakage was maintained at a low level in the dark, even at high HMON@CuS/Gd_2_O_3_ concentrations. However, HMON@CuS/Gd_2_O_3_ under NIR irradiation induced higher levels of LDH leakage in a concentration-dependent manner (Figure 4(B)). These results suggested that HMON@CuS/Gd_2_O_3_ provides promising destruction of the cell membrane structure, resulting in the release of lactate dehydrogenase from the cytoplasm into the culture medium under NIR irradiation.

Similarly, the abovementioned results were also confirmed by live-dead cell staining. Regarding cell growth, compared with cells in the saline group, saline under NIR irradiation group, and HMON@CuS/Gd_2_O_3_ in the dark group, the amounts of red spots (dead cells) significantly increased in HMON@CuS/Gd_2_O_3_ in the NIR irradiation group (Figure 4(C,D)). These results demonstrated that HMON@CuS/Gd_2_O_3_ can achieve effective therapeutic efficacy under NIR irradiation.

#### Lysosomal disruption based antitumour effect

**Figure 5.**
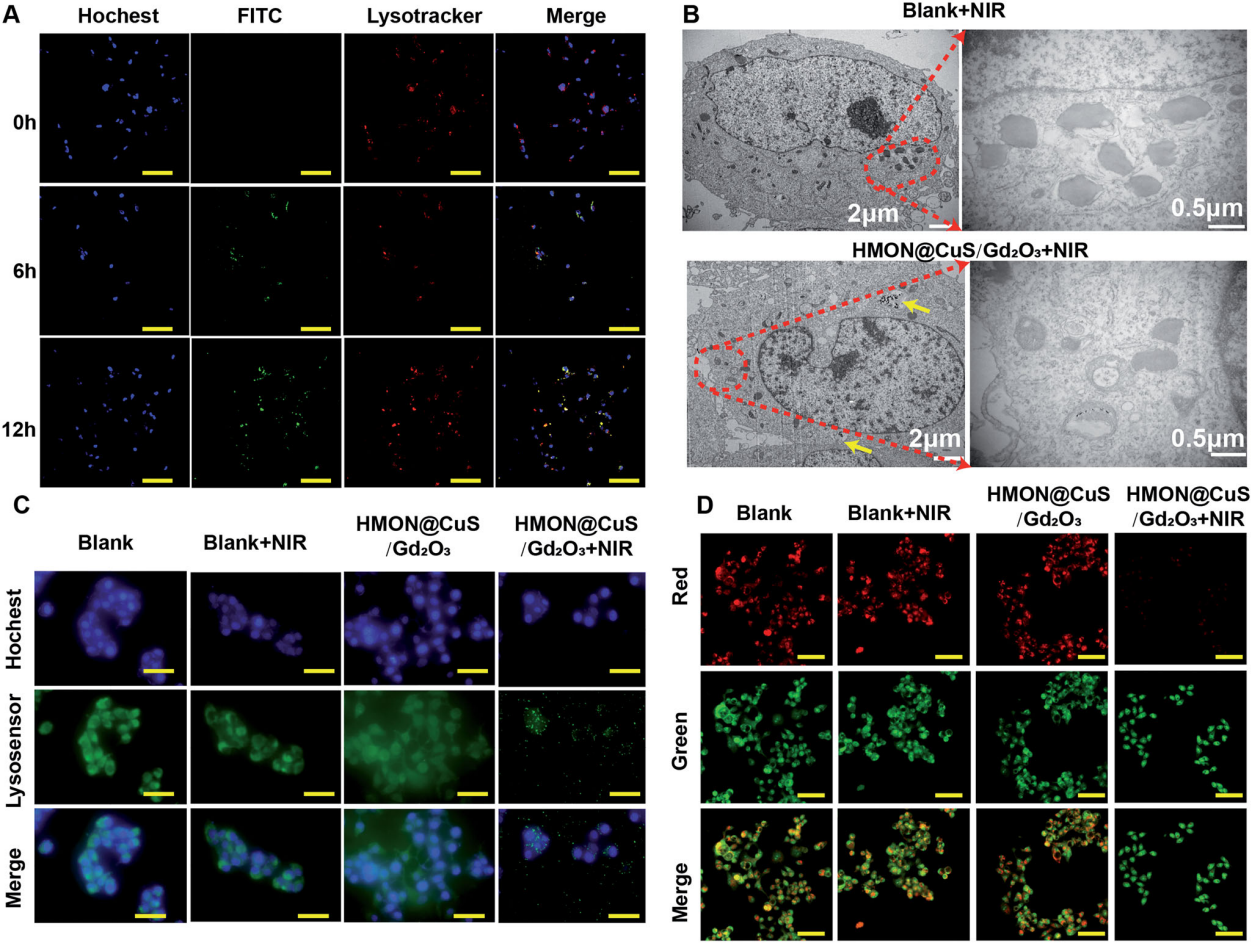
Antitumour mechanism induced by HMON@CuS/Gd_2_O_3_ plus NIR based on lysosomal disruption. (A) Confocal imaging of SKOV-3 cells incubated with FITC-labelled HMON@CuS/Gd_2_O_3_. At the designated incubation time, the cells were costained with LysoTracker Deep Red and Hoechst 33342 for imaging. Scale bar, 100 μm. (B) TEM images of SKOV-3 cells treated with HMON@CuS/Gd_2_O_3_ plus NIR irradiation. (C) Fluorescence images of SKOV-3 cells stained with LysoSensor Green DND-189 (scale bar, 30 μm). (D) Confocal imaging of SKOV-3 cells preincubated with HMON@CuS/Gd_2_O_3_ plus NIR irradiation followed by staining with acridine orange (AO). Scale bar, 50 μm.

Herein, a cell-permeant 2′,7′-dichlorofluorescin diacetate (DCFH-DA) probe was used to measure the intracellular ROS level. As presented in Figure S3, the control group exhibited little fluorescence, even in the presence of NIR irradiation, and negligible fluorescence was also observed in the HMON@CuS/Gd_2_O_3_ groups without NIR irradiation. Once exposed to the 808 nm laser irradiation, the HMON@CuS/Gd_2_O_3_ group showed higher DCF fluorescence, indicating that HMON@CuS/Gd_2_O_3_ could generate ROS and be used for PDT. To further investigate the mechanism of therapeutic efficacy of HMON@CuS/Gd_2_O_3_ under NIR irradiation, SKOV-3 cells were incubated with FITC-labelled HMON@CuS/Gd_2_O_3_ for 6 and 12 h. Subsequently, cells were stained with LysoTracker (Chen et al., 2012) (a dye specific for lysosomes with red fluorescence emission) and observed by CLSM. Compared with cells in the control group, the green fluorescence of HMON@CuS/Gd_2_O_3_ overlapped with the red fluorescence of the lysosome without NIR irradiation, and the yellow spots increased significantly over time (Figure 5(A)), indicating that HMON@CuS/Gd_2_O_3_ is located in the lysosome after internalization into SKOV-3 cells. TEM images demonstrated that lysosomes showed differences in shape and size, and lysosome vacuolation was occasionally observed after NIR laser irradiation (Figure 5(B)).

Additionally, after incubation with LysoSensor Green DND-189, the green fluorescence of the lysosome almost disappeared under NIR irradiation relative to the saline group, saline under NIR irradiation group, and HMON@CuS/Gd_2_O_3_ in the dark group, indicating that the skeleton structure of the lysosome was almost completely destroyed by HMON@CuS/Gd_2_O_3_ under NIR irradiation (Figure 5(C)). Furthermore, in this study, we performed an AO staining assay to evaluate the integrity of the lysosomal membranes (Zhang et al., 2014). Similar to the blank group, the cells treated with HMON@CuS/Gd_2_O_3_ showed red fluorescent dots, indicating that the lysosomal membranes were integrated (Figure 5(D)). However, negligible red fluorescent dots were observed in the cells incubated with HMON@CuS/Gd_2_O_3_ plus NIR irradiation, demonstrating that the phototherapeutic effect can rupture the lysosomal membrane structure and increase lysosomal membrane permeation. Thus, lysosomal disruption plays a critical role in the antitumour effect of HMON@CuS/Gd_2_O_3_.

#### *In vivo* multi-mode imaging of HMON@CuS@Gd_2_O_3_

**Figure 6.**
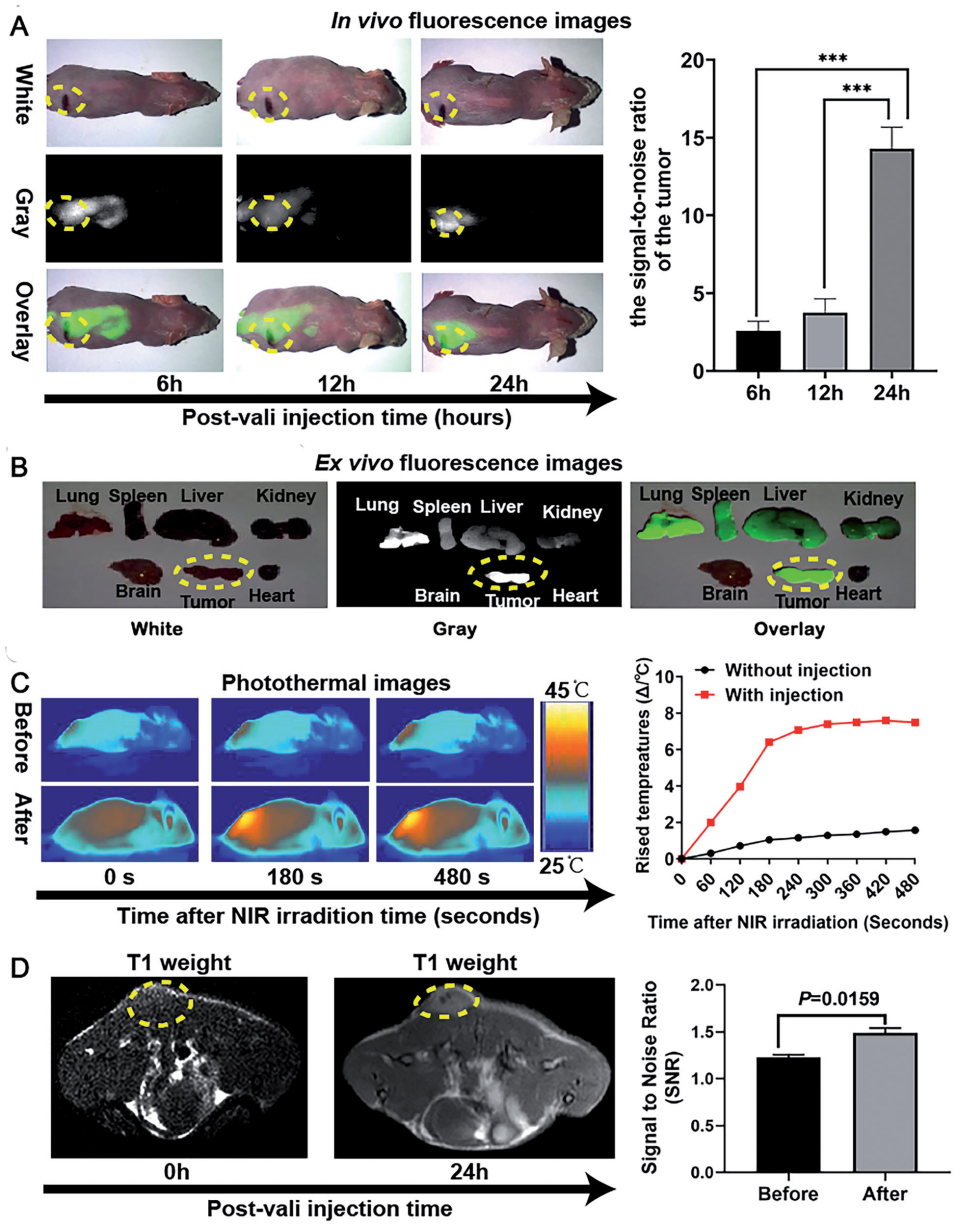
*In vivo* multimode imaging behaviors of HMON@CuS/Gd_2_O_3_ NPs in SKOV-3 cells. (A&B) *In vivo* and *ex vivo* fluorescence images of tumor-bearing mice 6, 12, and 24 h after treatment with HMON@CuS/Gd_2_O_3_ NPs, *** *p* < .001. (C) IRT images (left) and statistical temperature changes (right) of tumor-bearing mice at the indicated time points. (D) MRI images (left) and statistical signal-to-noise ratio (right) of tumor-bearing mice.

The fluorescence, MRI, and infrared thermal (IRT) multimodality imaging functionalities of HMON@CuS/Gd_2_O_3_ were evaluated in SKOV-3 tumor-bearing mice. Before fluorescence imaging, DIR, a near-infrared carbocyanine dye (Qu et al., 2020), was encapsulated into HMON@CuS/Gd_2_O_3_ NPs (HMON@CuS/@Gd_2_O_3_-DIR). Fluorescence images were acquired using a white-light and near-infrared dual-channel image-guided device (DIGITAL PRECISION MEDICINE Company, Beijing, China). Fluorescence signals were observed at the tumor site 6, 12, and 24 h after injecting HMON@CuS/Gd_2_O_3_ NPs intravenously into tumor-bearing mice, with maximal fluorescence intensity after 24 h (Figure 6(A)). Twenty-four hours later, the excised heart, liver, spleen, lung, kidney, brain, and tumor tissues were further investigated, and the fluorescence intensity of tumor tissue was significantly higher than that of other tissues (Figure 6(B)). These results suggested that HMON@CuS/Gd_2_O_3_ NPs mainly accumulate in tumor tissue and reach a peak at 24 h; therefore, 24 h postinjection was an optimal therapeutic time window for therapy treatment *in vivo*. Fluorescence signals in the lung, spleen, liver, and kidney were also observed in the mice, indicating that the constructed NPs based on HMONs undergo reticuloendothelial system uptake and renal excretion. The biocompatibility of NPs based on HMONs was tested in our earlier reported studies (Guo et al., 2020).

Subsequently, the IRT imaging abilities of HMON@CuS/Gd_2_O_3_ were further investigated in SKOV-3 tumor-bearing mice to evaluate the hyperthermia efficacy *in vivo*. The temperature of the tumor site was evaluated before intravenous tail injection of NPs and 24 h postinjection. The minimal tumor temperature only increased by approximately 2 °C under NIR laser irradiation (.5 W/cm^2^; 8 min) before injection. Although the minimal tumor temperature rose approximately 8 °C 24 h postinjection, the temperature rose rapidly to 42 °C within 180 s and was maintained at 43–445 °C in the following 300 s. These results indicated that HMON@CuS/Gd_2_O_3_ exhibits a mild PTT effect and good photothermal stability under NIR irradiation (.5 W/cm^2^), a feature that may be much more attractive for clinical phototherapeutic treatment.

Furthermore, the *in vivo* T1-weighted MR imaging capacity of HMON@CuS/Gd_2_O_3_ was evaluated in SKOV-3 tumor-bearing mice using a 3.0 T Philips Ingenia MRI scanner. The intensity of the T1-weighted MR signal at the tumor site significantly increased 24 h after intravenous administration, and the signal-to-noise ratio (SNR) of the tumor 24 h after injection was 1.5 times higher than that before injection, validating the potential of HMON@CuS/Gd_2_O_3_ for T1-weighted MR imaging. These results consistently confirmed the tumor targeting capacity of HMON@CuS/Gd_2_O_3_.

Hence, the verified fluorescence, IRT, and MR imaging functionalities of HMON@CuS/Gd_2_O_3_ NPs synergistically provide various imaging features for precise cancer diagnostics and treatment. This multifunctional nanoplatform integrates the characteristics of high sensitivity, deep penetration and real-time navigation of fluorescence imaging, high spatial resolution, anatomical resolution, and good soft tissue resolution of MR imaging, as well as the tumor ablation characteristics of IRT.

#### *In vivo* antitumour of HMON@CuS/Gd_2_O_3_

**Figure 7.**
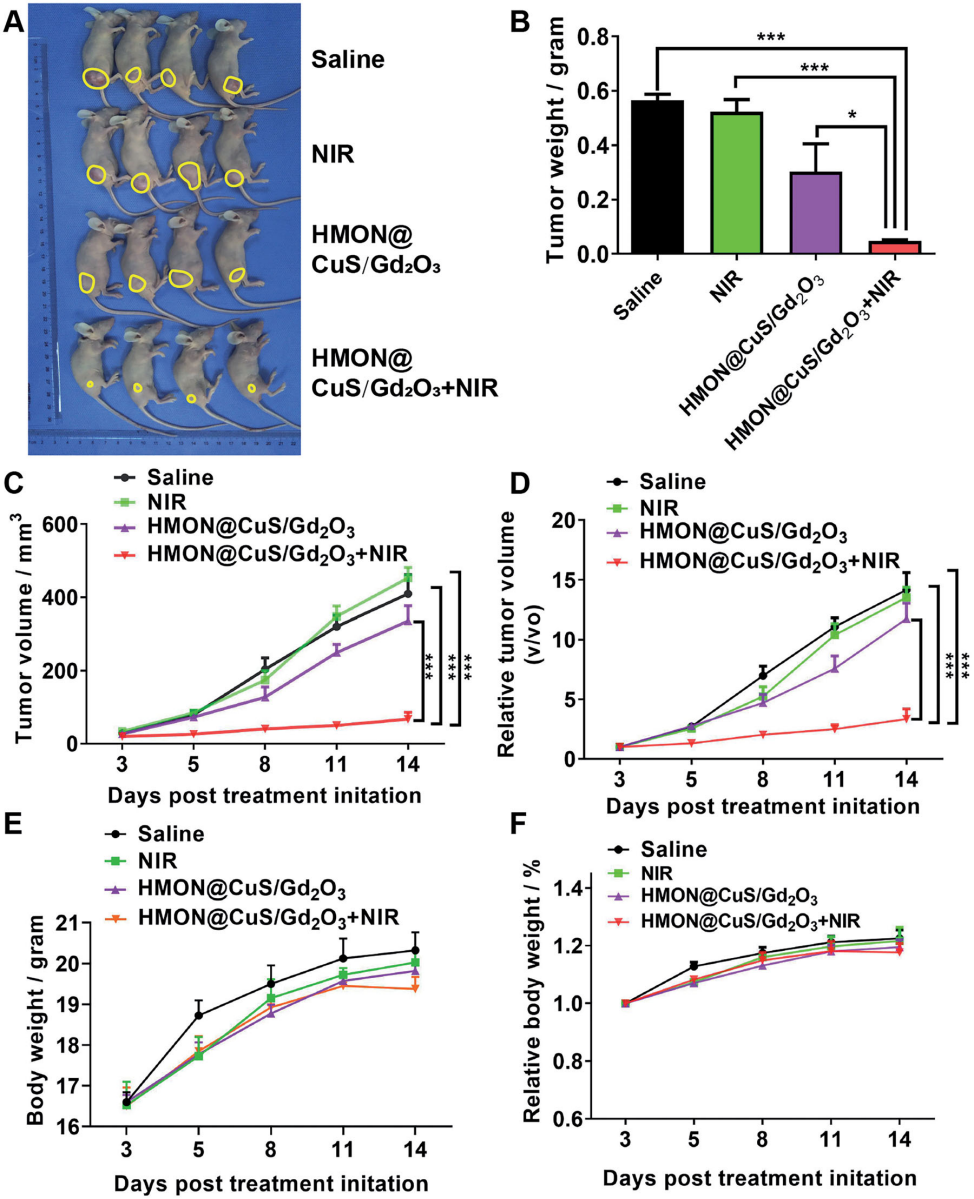
*In vivo* antitumour effects. (A) Images of tumors derived from mice after treatment with saline, saline plus NIR, HMON@CuS/Gd_2_O_3_, and HMON@CuS/Gd_2_O_3_ plus NIR. (B) Tumor weight of tumors derived from mice. (C&D) Tumor volume and relative tumor volume of tumors derived from mice. (D&E) Body weight and relative body weight of tumors derived from mice. *N* = 4, *p < .05, ***p < .001.

To further analyze the *in vivo* antitumour potency, tumor-bearing mice were randomly divided into four groups: saline (negative control), saline under NIR irradiation (.5 W/cm^2^; 8 min), HMON@CuS/Gd_2_O_3_, and HMON@CuS/Gd_2_O_3_ under NIR irradiation. The tumor suppression efficacy was quantitatively estimated by measuring the tumor size and tumor weight ( (Figure 7). The mice in the saline group and saline under NIR irradiation group showed rapid tumor growth (almost 15-fold increase in the tumor volume on the 14^th^ day), indicating that NIR irradiation alone does not play an antitumour role. The mice in the HMON@CuS/Gd_2_O_3_ group without NIR irradiation showed a similar tumor growth rate to those in the saline group, verifying that HMON@CuS/Gd_2_O_3_ alone has very limited antitumour efficacy. Interestingly, the tumor-bearing mice in the HMON@CuS/Gd_2_O_3_ under NIR irradiation group exhibited a marked tumor inhibition effect, and the tumor growth inhibition rate was approximately 80% compared with that in the control group. Additionally, no significant differences were observed in the body weight among these four groups, indicating that those treatments were well tolerated by the mice. Taken together, the results revealed that the photothermal therapy induced by HMON@CuS/Gd_2_O_3_ plus NIR irradiation efficiently kills tumor cells with fewer side effects, demonstrating that HMON@CuS/Gd_2_O_3_ NPs have promising applications for ovarian cancer treatment.

## Conclusion

In summary, a new nanoplatform for endolysosomal escape and multimodal imaging was successfully constructed. The as-prepared NPs exhibit mild-temperature photothermal therapeutic effects under mild NIR irradiation (.5 W/cm^2^), followed by lysosome vacuolation, disruption of lysosomal membrane integrity, and finally inhibition of the cell proliferation ability of ovarian cancer. Additionally, HMON@CuS/Gd_2_O_3_ have enhanced T1 MR imaging, FL imaging, and IRT imaging capacities, which can realize multimodal imaging-guided precision phototherapy. Taken together, the findings suggest that these well-synthesized nanoplatforms are promising anticancer agents to treat ovarian cancer and show great potential for biomedical applications.

## Supplementary Material

Supplemental Material

## Data Availability

The data of this study are available from the corresponding author upon reasonable request.
