# Supplementary material for: Biodegradable hollow mesoporous organosilica nanotheranostics (HMONs) as a versatile platform for multimodal imaging and phototherapeutic-triggered endolysosomal disruption in ovarian cancer
Source: Drug Deliv. 2021 Dec 30;29(1):161–73. doi: 10.1080/10717544.2021.2021322 (PMC8725973; doi:10.1080/10717544.2021.2021322)
Supplement: Supplemental Material [file IDRD_A_2021322_SM8023.zip › Supplementary Material.docx]

*Supporting Information*

*Title: Biodegradable hollow mesoporous organosilica nanotheranostics (HMONs) as a versatile platform for multimodal imaging and phototherapeutic-triggered endolysosomal disruption in ovarian cancer*


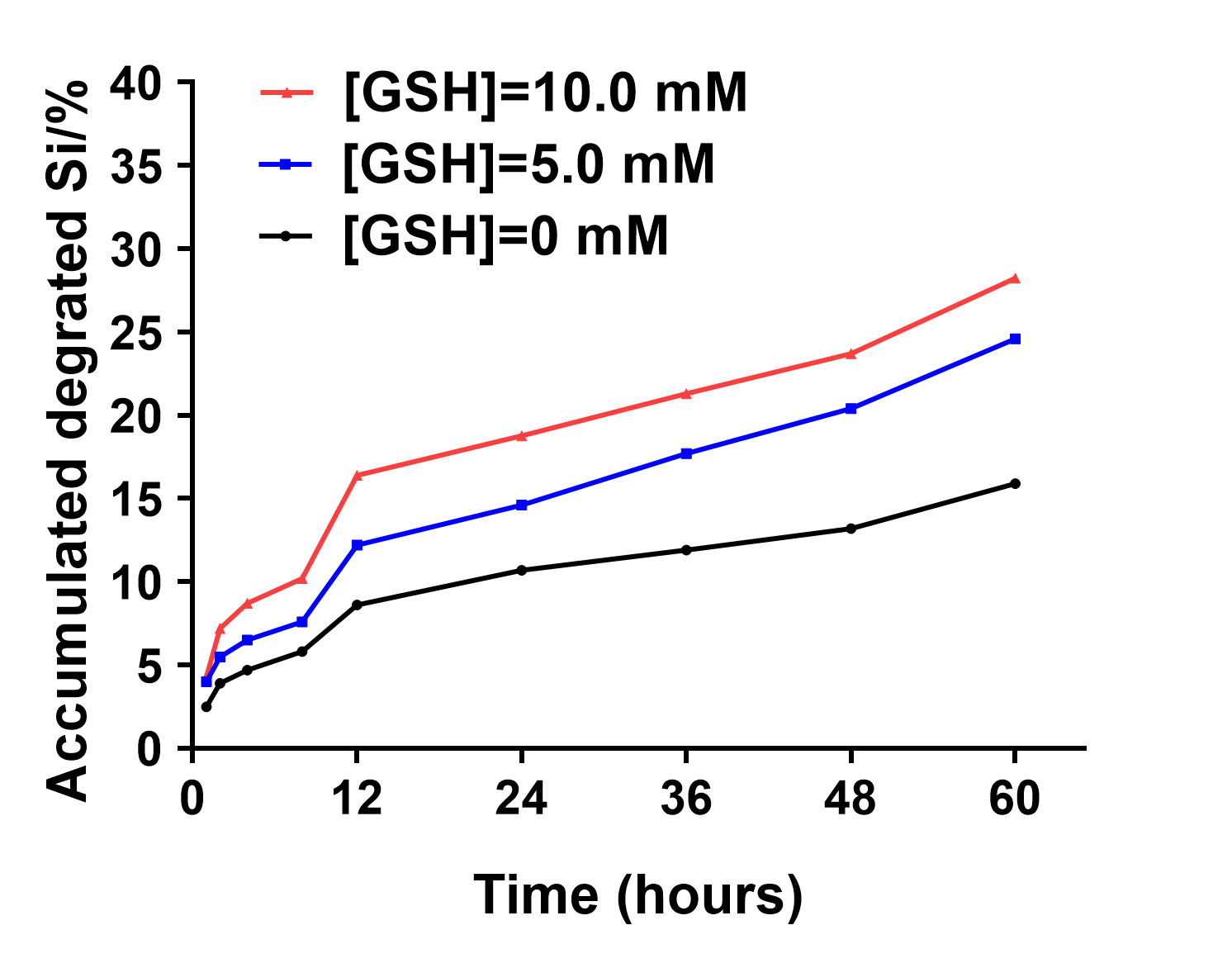


***Figure S1***. Cumulative Si ions release from HMON@CuS/Gd_2_O_3_ NPs at different gluthathione (GSH) concentrations (0, 5, and 10 mM).


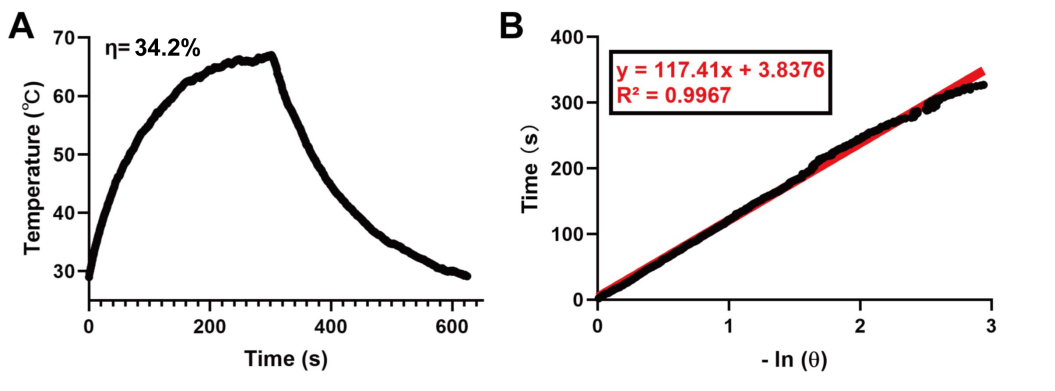


***Figure S2***. (A) Photothermal performance of HMON@CuS/Gd_2_O_3_ nanoparticles under NIR irradiation (0.5 W/cm^2^, 5 min), with the laser cut off when the temperature became stable. (B) Fitting curve of the time versus -ln(θ).


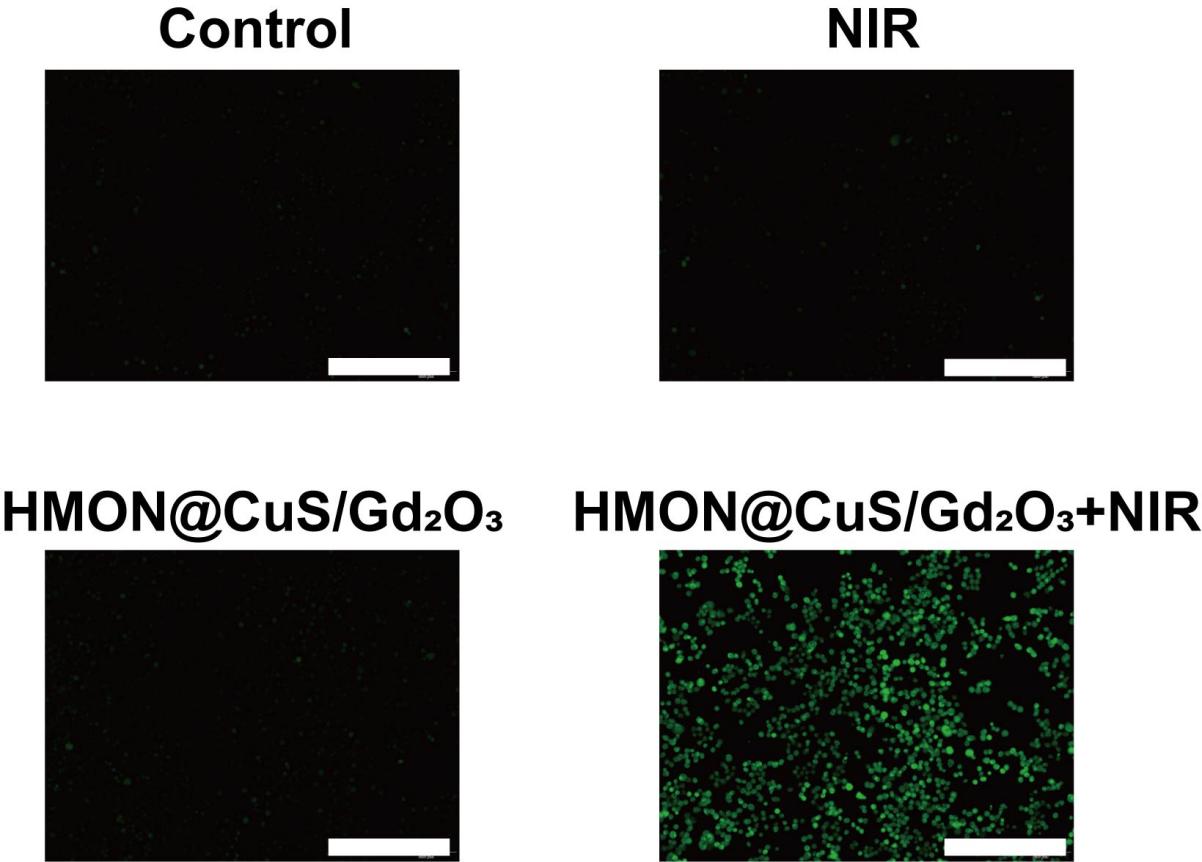


***Figure S3.*** Images of ROS in treated SKOV-3 cells. Scale bar: 400 μm.
