# Supplementary figures and images for: Biodegradable hollow mesoporous organosilica nanotheranostics (HMONs) as a versatile platform for multimodal imaging and phototherapeutic-triggered endolysosomal disruption in ovarian cancer
Source: Drug Deliv. 2021 Dec 30;29(1):161–73. doi: 10.1080/10717544.2021.2021322 (PMC8725973; doi:10.1080/10717544.2021.2021322)

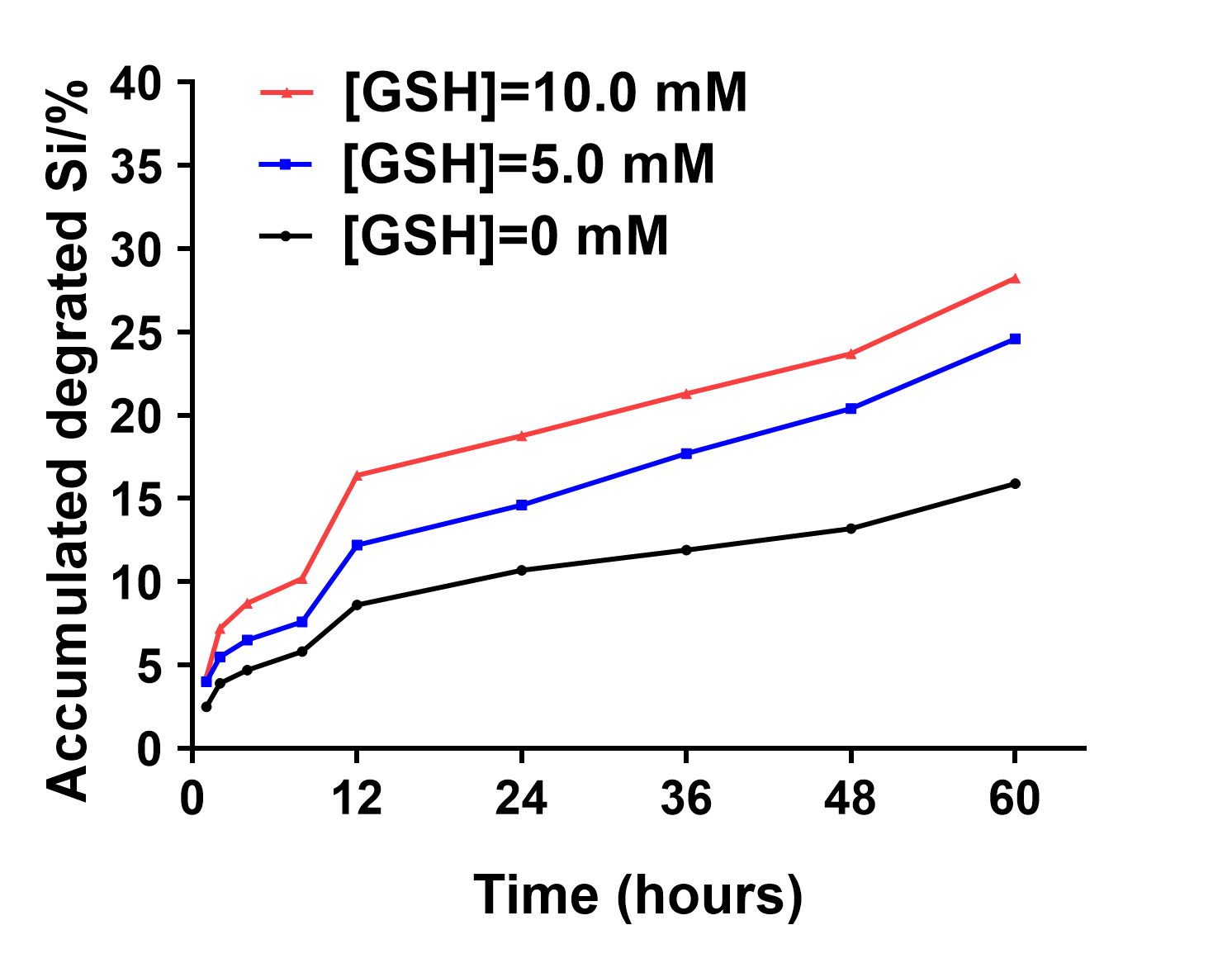

Supplement: Supplemental Material [file IDRD_A_2021322_SM8023.zip › Figure S1.tif]
